# Engineering nanomedicines through boosting immunogenic cell death for improved cancer immunotherapy

**DOI:** 10.1038/s41401-020-0400-z

**Published:** 2020-04-21

**Authors:** Jing Gao, Wei-qi Wang, Qing Pei, Megan S. Lord, Hai-jun Yu

**Affiliations:** 1 Key Laboratory of Drug Research & Centre of Pharmaceutics, Shanghai Institute of Materia Medica, Chinese Academy of Sciences, Shanghai, 201203 China; 2 Peking University Shenzhen Institute, Shenzhen, 518055 China; 3 School of Pharmacy, Nantong University, Nantong, 226001 China; 4 Graduate School of Biomedical Engineering, University of New South Wales, Sydney, NSW 2052 Australia

**Keywords:** cancer immunotherapy, immunogenic cell death, immunosuppressive tumor microenvironment, nanomedicine, drug delivery systems

## Abstract

Current cancer immunotherapy has limited response rates in a large variety of solid tumors partly due to the low immunogenicity of the tumor cells and the immunosuppressive tumor microenvironment (ITM). A number of clinical cancer treatment modalities, including radiotherapy, chemotherapy, photothermal and photodynamic therapy, have been shown to elicit immunogenicity by inducing immunogenic cell death (ICD). However, ICD-based immunotherapy is restricted by the ITM limiting its efficacy in eliciting a long-term antitumor immune response, and by severe systemic toxicity. To address these challenges, nanomedicine-based drug delivery strategies have been exploited for improving cancer immunotherapy by boosting ICD of the tumor cells. Nanosized drug delivery systems are promising for increasing drug accumulation at the tumor site and codelivering ICD inducers and immune inhibitors to simultaneously elicit the immune response and relieve the ITM. This review highlights the recent advances in nanomedicine-based immunotherapy utilizing ICD-based approaches. A perspective on the clinical translation of nanomedicine-based cancer immunotherapy is also provided.

## Introduction

Cancer immunotherapy has changed the paradigm of clinical cancer therapy by demonstrating that boosting the systemic immune response can lead to tumor regression [1, 2]. Despite the potential of immunotherapy to cure malignant tumors, the clinical application of current cancer immunotherapy has been hampered by several challenges, including insufficient intratumoral infiltration of cytotoxic T lymphocytes (CTLs) and the immunosuppressive tumor microenvironment (ITM) [3, 4]. Solid tumors with low immunogenicity are defined as “cold tumors,” and they differ from solid tumors with high immunogenicity, which are termed “hot tumors” [5]. Immune checkpoint blockade (ICB) therapy has been extensively investigated to overcome ITM [6, 7], while many other therapeutic modalities, including cancer vaccines, cytokine therapy and adoptive T-cell transfer therapy, have also been exploited for cancer immunotherapy [8–10].

**Fig. 1:**
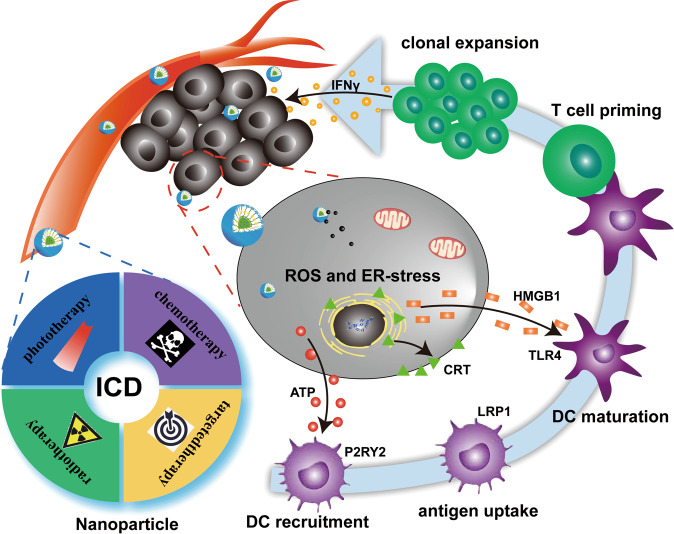
Schematic of nanoparticle-mediated ICD for synergistic cancer immunotherapy. After overcoming pathophysiological barriers, multifunctional nanoparticles composed of ICD inducers and immune inhibitors induce tumor cell death via immunogenic pathways. The DAMPs released by dying cells play a crucial role in modulating the immunogenicity of the tumor microenvironment, including promoting the recruitment of APCs through tumor cell-derived secreted ATP and HMGB1 and facilitating DC maturation. Subsequently, activated DCs prime T cells, enabling CTLs to kill tumor cells via IFN-γ-dependent mechanisms.

Immunogenic cell death (ICD) is an alternative approach that activates antitumor immunity to eradicate tumor cells [11]. Tumor cells undergoing ICD secrete distress signals through molecules called damage-associated molecular patterns (DAMPs), including calreticulin (CRT), adenosine triphosphate (ATP) and high-mobility group box 1 (HMGB1), to activate the immune response [12–14]. These DAMPs facilitate the cancer immunity cycle. For instance, the presentation of CRT, an endoplasmic reticulum (ER) chaperone, on the tumor cell surface, along with ATP release, induce antigen presenting cells (APCs), including dendritic cells (DCs), to phagocytose tumor cells [15]. In addition, the release of HMGB1 from dying tumor cells promotes interactions of toll-like receptor 4 (TLR-4), a pattern recognition receptor that signals through the TLR-4-MyD88 axis on DCs, that elicit potent immunostimulatory effects [16]. Exogenous HMGB1 also facilitates the maturation of DCs, enabling their trafficking to the lymphnodes for antigen presentation and T cells priming [17]. Thereafter, activated CTLs recognize tumor cells and induce tumor regression by secreting cytokines, including interferon-γ (IFN-γ), tumor necrosis factor-α and interleukin-6 [18–21] (Fig. 1).

Recently, it was demonstrated that chemotherapeutics (e.g., oxaliplatin (OXA), doxorubicin (DOX), and mitoxantrone) and tyrosine kinase inhibitors, such as crizotinib elicit antitumor immunity by inducing the immunogenic death of tumor cells [22–26]. Furthermore, several physical stimulis also induce ICD. For example, photodynamic therapy (PDT), irradiation and photothermal therapy (PTT) trigger reactive oxygen species (ROS) generation to induce the intracellular ER stress that initiates the ICD cascade and DAMPs release [27–30].

Despite the promising potential of ICD-based approaches for cancer therapy, inadequate induction of ICD by conventional approaches has hampered clinical translation. Shortcomings of conventional approaches include insufficient circulation time, limited intratumoral accumulation and retention, restricted tumor cellular uptake and slow release in tumor cells [31, 32]. Moreover, on-target but off-tumor toxicity of the ICD inducers has also limited the clinical translation of ICD-based cancer therapy approaches [33, 34].

To overcome these challenges with conventional ICD approaches, nanoparticle-based drug delivery systems with tunable size and surface properties offer unique advantages for ICD-based cancer therapy (Fig. 1). Nanoparticles enable targeted drug accumulation at the tumor site, enhance tissue penetration and increase cellular uptake, as well as trigger the tailored release of ICD inducers [35–40]. Furthermore, nanoparticle-based drug delivery systems may also deliver other immunomodulatory agents to diminish the ITM [41]. Thus, nanoparticle-based ICD approaches offer the potential of enhancing ICD and reversing the ITM in a controllable manner.

This review discusses recent advances in nanoparticle-mediated ICD and focuses on the effects induced by certain drugs and physical stimuli. Novel insights into engineering nanoparticles to enhance their antitumor efficacy utilizing a combination of ICD and ITM reprogramming are also provided.

## Therapeutic modalities that induce ICD

### Chemotherapy

**Fig. 2:**
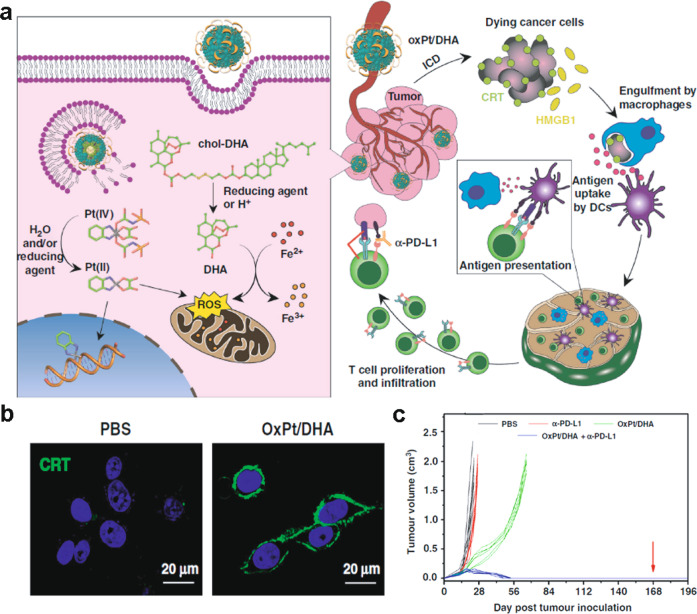
Schematic of OXA-loaded DHA NCPs (OxPt/DHAs) with synergistic antitumor activity by inducing ICD. **a** Chemical structure and synergistic mechanism of tumor inhibition; **b** Confocal images of cell surface localization of CRT upon exposure to OxPt/DHAs; **c**. Tumor growth over time with OxPt/DHAs or combination therapy with checkpoint blockade (anti-PD-L1) in a mouse model of CT26 colorectal tumor. Adapted with permission from [44]. Copyright (2019) Nature Publishing Group.

Chemotherapy is a main stream clinical cancer treatment, and recent evidence suggests that several chemotherapeutics have the capacity to induce ICD. Nevertheless, the potency of the ICD effect is reduced because of insufficient tumor accumulation and/or adverse nonspecific cytotoxic effects [42]. Systemic delivery of chemotherapies with nanosized drug delivery systems is a promising strategy to address these challenges because they have advantages in terms of sufficient circulation time, increased intratumoral accumulation and retention, highly efficient uptake by tumor cells and precise release at tumor tissues, compared with these features of chemotherapeutic agents administered alone. For instance, OXA encapsulated in a self-assembling methoxy poly(ethylene glycol)-block-poly(*D*, *L*-lactide-co-glycolide) (mPEG-*b*-PLGA) nanocarrier enhanced the induction of ICD to a greater extent than did OXA delivered alone via the promotion of DAMPs, enhanced maturation of DCs and elevated levels of intratumor infiltrating CTLs [43]. In addition, OXA encapsulated in mPEG-*b*-PLGA was more effective in inhibiting tumor growth in both immunocompetent and immunodeficient mice than was OXA. Similar results were also reported for OXA-loaded iron oxide nanoparticles that induced ICD by increasing ER stress. Self-assembled nanoscale coordination polymer (NCP) core-shell nanoparticles containing OXA in the core and dihydroartemesinin (DHA) in the shell conjugated to cholesterol via disulfide bonds were cleaved by glutathione (GSH) in tumor cells to release the chemotherapeutic and exert ICD effects (Fig. 2a) [44]. Both OXA and DHA induce oxidative stress (Fig. 2b) and were found to synergistically enhance these effects through delivered OXA-loaded NCP nanoparticles. In addition, these particles promoted intratumoral infiltration of CTLs and activated the immune system for tumor regression (Fig. 2c).

DOX has been investigated for the ICD induction of tumor cells [45]. For example, chimeric polypeptide-conjugated DOX nanoparticles promoted IFN-γ secretion, facilitated the intratumoral infiltration of CD8^+^ T cells by ICD induction and more efficiently reduced tumor size than did DOX administered alone [46]. In addition to OXA and DOX, mitoxantrone has also been reported to activate antigen release via the ICD cascade [47]. A promising strategy involves combining chemotherapeutic drugs with immune adjuvants to enhance host-specific antitumor immunity. Among the various adjuvants, CpG oligonucleotides, which function as TLR-9 agonists, have been widely investigated for immunotherapy applications [48]. For example, CpG-loaded lipid-polymer hybrid nanoparticles containing mitoxantrone induced an antigen-specific CD8^+^ T cell response with a higher level of ICD than did mitoxantrone administered alone and significantly inhibited tumor growth in a CT26 tumor model [49].

In addition to CpG oligonucleotides, interleukin-2 (IL-2) is an immune adjuvant used for activating CTLs and natural killer (NK) cells [50]. Thermosponge nanoparticles (TSNs) composed of mPEG-PLGA loaded with paclitaxel (PTX) in the core and IL-2 in the shell (PTX/IL-2-TSNs) reduced tumor growth and metastasis in a murine melanoma model to a greater extent than did either TSNs loaded with PTX or IL-2, demonstrating the benefit of combining chemotherapy with an immune adjuvant [51].

### Radiotherapy

Radiotherapy is another main stream method to treat local solid tumors by destroying DNA double strands [52]. Moreover, several studies have demonstrated that radiotherapy induces the release of tumor-associated antigens (TAAs) that trigger antitumor immune responses [53, 54]. For example, PLGA-based core-shell nanoparticles have been shown to enhance the efficacy of radiotherapy [55]. A double emulsion strategy was used to encapsulate catalase (Cat) inside the aqueous core and to integrate imiquimod (also known as R837, a potent TLR-7 agonist) into the hydrophobic shell of PLGA (PLGA-R837@Cat). Cat was incorporated because it decomposes H_2_O_2_ into H_2_O and O_2_ to reduce hypoxia-associated radiation resistance. PLGA-R837@Cat induced ICD after X-ray irradiation in a CT26 murine tumor model, as demonstrated by the cell surface localization of CRT and increased DC maturation. These nanoparticles, in combination with X-rays, reduced the size of the primary tumors. In addition, the combination of PLGA-R837@Cat with clinically approved CTLA-4 checkpoint blockade therapy inhibited tumor metastasis and prevented cancer recurrence.

### PTT

PTT utilizes a photoabsorber to convert photoenergy into thermal energy upon laser irradiation [56]. The hyperthermic effect induced by PTT destroys tumor cells by disrupting the cell membrane, denaturing proteins and inducing DNA damage [57]. In addition, PTT induces immune responses by releasing TAAs and other immunostimulatory molecules [58, 59]. To take advantage of PTT-induced ICD, Prussian blue nanoparticles combined with PTT administered at temperatures between 63.3 and 66.4 °C induced a robust ICD-mediated antitumor effect and long-term survival in a neuroblastoma tumor model [60]. In addition, magnetic iron oxide nanoparticles coated with myeloid-derived suppressor cell membrane (MNPs@MDSC) have been reported to accumulate in tumor tissues and act as PTT agents to induce local hyperthermia and trigger ICD, as demonstrated by increased tumor cell surface CRT expression and delayed tumor growth [61]. PTT-based cancer immunotherapy is limited by reduced laser penetration, insufficient activation of the ICD effect and an extant ITM. To address these challenges, extensive efforts have been made to boost PTT-based ICD. For instance, Ma et al. utilized near-infrared II (NIR-II) light with high tissue penetration ability to promote PPT-mediated cancer immunotherapy. An in vivo antitumor study demonstrated that NIR-II laser-based PTT-induced DAMP release in deep tumor tissue, eliciting DC maturation at high efficiency, and facilitated intratumoral infiltration of CTLs [62].

### PDT

PDT destroys tumors by a combination of light, oxygen and photosensitizers (PS) due to the generation of highly toxic ROS [63]. Once activated by a laser emitting a specific wavelength, the generated cellular ROS inhibited tumor growth by destroying the vasculature within the tumor, specifically by damaging organelles and promoting the secretion of proinflammatory cytokines. PDT has been shown to enhance tumor immunogenicity by inducing ICD via the upregulation of tumor cell surface antigens and activation of CTLs [64]. Hence, PDT is a promising therapeutic modality for eliminating residual tumor cells [65]. However, PSs are generally hydrophobic molecules, and therefore, their photoactivity is compromised by their poor solubility in biological media and low accumulation at the tumor site [66].

Nanoparticle-based delivery systems offer enhanced delivery of PSs to tumors and reduced systemic toxicity [67]. A core-shell nanoparticle prepared on the basis of the coordination between Zn^2+^ and photoactive pyrophosphate was coated with the PS pyrolipid (ZnP-Pyro) and showed prolonged circulation in blood, high accumulation within tumors and high cell surface CRT expression [68]. ZnP-Pyro promoted ROS-induced ICD, suppressed primary tumors and inhibited lung metastasis. An alternative approach to achieve high tumor specificity is based on using the NK cell membrane to enhance tumor targeting and promote M1-type macrophage polarization [69]. A porphyrin-like PS was loaded onto the NK cell membrane (NK-NP) and achieved prolonged circulation in blood and induced ICD. Both the number of matured DCs and the ratio of CD8^+^ T cells in distal tumors were significantly increased after laser treatment compared with the number and ratio of the control group. NK-NPs not only eliminated the growth of the primary tumor but also prevented the progression of the distant tumor.

The antitumor efficacy of PDT is also limited by the hypoxic tumor environment [70, 71]. Thus, some approaches have explored oxygen nanocarriers, such as hemoglobin (Hb), hybridized with human serum albumin via disulfide bonds and loaded with the PS chlorine e6 (C@HPOC) [72]. The C@HPOC supplied oxygen to the tumor and upon laser irradiation increased ROS, as well as promoted the release of HMGB1 and ATP and the cell surface expression of CRT. Similarly, core-shell gold nanocages coated with manganese dioxide (MnO_2_) nanoparticles were found to degrade in acidic tumor environments and produce oxygen that promoted the antitumor immunity response, while nanoparticles without the ability to produce oxygen did not elicit a similar antitumor immune response [73]. Thus, approaches that supply oxygen to the tumor offer a new way to enhance antitumor activity and reverse the ITM.

ER targeting to trigger ER stress is another approach to enhance the ICD efficacy of PDT [74, 75]. Gold nanospheres containing ER-targeting pardaxin (FAL) peptides and indocyanine green (ICG) (FAL-ICG-HAuNSs) were explored for both PDT- and PTT-mediated tumor therapy [76]. These particles were found to accumulate in the ER. When delivered together with FAL-modified Hb liposomes (FAL-Hb lipos) and exposed to near-infrared (NIR) radiation, high levels of ROS were generated compared with those generated by the control. Delivery of this particle combination together with NIR irradiation showed effective tumor growth inhibition and increased survival rates in a CT26 tumor model.

### Combinationtherapy

Cancer monotherapy is limited by tumor heterogenicity and the complex tumor immune microenvironment. Hence, combination therapies may offer some advantages [77, 78]. For example, a chemotherapeutic and PS-loaded self-assembling triblock copolymer composed of polyethylene glycol-poly(methyl methacrylate-co-2-amino ethyl methacrylate (thiol/amine))-poly(2-(dimethylamino)ethyl methacrylate) (PEG-P(MMA-co-AEMA (SH/NH_2_)-PDMA)) has been explored for its anticancer activity [79]. These particles were used to encapsulate a low dose of DOX and a photoactive agent, 2-(1-hexyloxyethyl)-2-devinylpyropheophorbide-a, that could be released via GSH-mediated cleavage for use in PDT [80]. These particles administered with laser irradiation increased the proportion of mature DCs in tumor draining lymph nodes and CD8^+^ T cells in MC38 colon adenocarcinoma cell tumors in mice [79]. In addition, the cell surface CRT expression was higher than that of the control, suggesting that the combination therapy exhibited enhanced efficacy compared with that of monotherapies.

## Approaches targeting the ITM

The tumor microenvironment plays a dominant role in tumor growth, recurrence and metastasis; however, the efficacy of traditional therapeutics is limited by the tumor microenvironment. Recently, immunosuppressive factors, including indoleamine 2,3-dioxygenase 1 (IDO-1) and ICB, have been investigated to overcome the barriers placed by the ITM.

### Inhibiting IDO-1 expression

The depletion of tryptophan (Trp) and accumulation of kynurenine (Kyn) by IDO-1 can hamper the antitumor immune response by suppressing the activity of CTLs and eliciting regulatory T cells [81]. Moreover, the IFN-γ secreted by CTLs has been demonstrated to promote the expression of IDO-1 [82]. Thus, the level of IDO-1 expression is key to modulating the ITM. Nanoparticle-based approaches have been explored, and a binary cooperative prodrug nanoparticle (BCPN) was designed to provide balance by codelivering an ICD inducer, OXA prodrug, and an IDO-1 inhibitor, NLG919, to augment the therapeutic efficacy of ICD [83]. BCPN released OXA and NLG919 in the acidic and reductant tumor microenvironment to promote ICD induction and IDO-1 inactivation [84, 85].

**Fig. 3:**
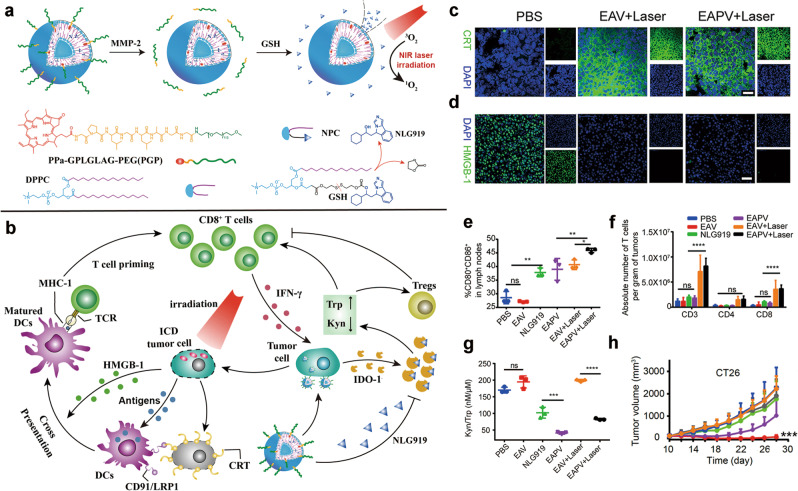
Schematic illustration of shedding prodrug vesicles for cancer immunotherapy. **a** The preparation procedure for enzyme-activatable prodrug vesicles (EAPV) designed to codeliver PEGylated PS and an IDO-1 inhibitor (NLG919); **b** The mechanisms by which EAPVs enhance antitumor efficacy via synergic triggering of ICD and combating IDO-1-mediated adaptive immune resistance; **c** Immunofluorescence image of CRT expressed on the surface of tumor cells in the CT26 tumor xenograft model (scale bar = 50 μm); **d** Immunofluorescence image of HMGB1 release from tumor cells in the CT26 tumor xenograft model (scale bar = 25 μm); **e** The ratio of mature DCs in tumor-draining lymph nodes; **f** The number of intratumor infiltrating T lymphocytes in tumor tissue; **g** Kyn to Trp molar ratio determined for the CT26 tumor tissue; and **h** Tumor growth curves of the CT26 xenograft tumors. Adapted with permission from [86]. Copyright (2019) American Chemical Society.

To achieve tumor-specific distribution and accumulation of the ICD inducer and IDO-1 inhibitor, enzyme-activatable prodrug vesicles (termed EAPVs), with the enzyme matrix metalloproteinase-2/9 (MMP-2/9), were codelivered with a PEGylated PS (e.g., PPa) and NLG919 (Fig. 3a, b) [86]. The prodrug vesicle was stable during blood circulation and accumulated in the tumor via the EPR effect. In the tumor, the PEG corona was cleaved via MMP-2-mediated degradation of the GPLGLAG peptide spacer, and NLG919 was released via the GSH-mediated reduction of the disulfide bonds. The EAPVs showed higher accumulation and retention in the tumors of a CT26 xenograft tumor model than they did in an MMP-2/9-insensitive control group, and the EAPVs showed increased ROS generation upon laser irradiation. The EAPVs also promoted CRT expression on the surface of the cell membrane and HMGB1 release, which subsequently supported DC maturation and recruited intratumorally infiltrating T lymphocytes to a greater extent than did the controls (Fig. 3c–f). Thus, EAPVs triggered PDT-mediated ICD as well as IDO-1 suppression, which supported tumor growth inhibition (Fig. 3g, h). Similarly, a synergistic nanoparticle containing a PS, protoporphyrin IX, and the IDO-1 inhibitor 1-methyltryptophan, conjugated via caspase-responsive peptides, also showed inhibition of the primary tumor and lung metastasis [87].

To precisely control drug release at the tumor site to promote antitumor immunogenicity and minimize systemic adverse effects, a light-inducible nanocargo (LINC) was designed for use in combination cancer immunotherapy [88]. A LINC was constructed with a GSH-responsive heterodimer of PPa and NLG919 and an ROS-labile PEGylated prodrug of OXA, which induced ICD and inhibited IDO-1 activity.

**Fig. 4:**
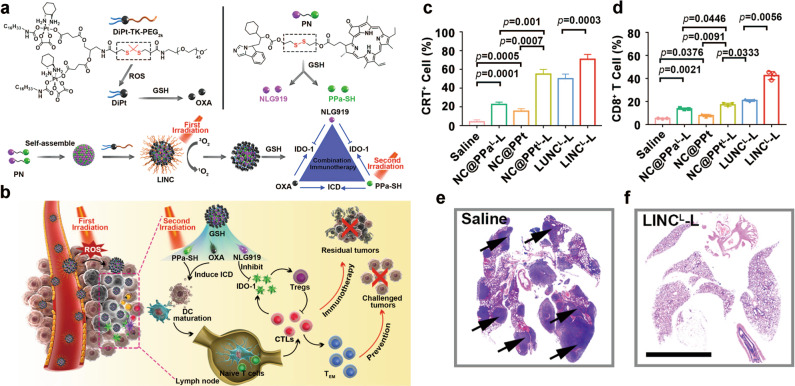
Schematic of LINC for cancer immunotherapy. **a** The chemical structures and self-assembly process of the LINC; **b** The mechanisms of NIR light-inducible nanoparticles that induce ICD and reprogram the immunosuppressive tumor microenvironment; **c** CRT expression; **d** Intratumoral infiltration of CD8^+^ T cells; and hematoxylin and eosin (H&E) staining of the lung sections collected from the (**e**) saline and (**f**) LINC^L^-treated mouse groups, respectively. Adapted with permission from [88]. Copyright (2019) John Wiley & Sons, Inc.

In a spatiotemporally controllable manner via dual laser irradiation steps (Fig. 4a). The first irradiation step promoted ROS generation and cleavage of the PEG corona following tumor accumulation to facilitate deep tumor penetration and intracellular uptake of the prodrug nanoparticles. Both OXA and NLG919 were restored inside the tumor cells via GSH-mediated reduction of the disulfide bond and OXA(IV) prodrug to trigger the immunogenic death of tumor cells. The second wave of laser irradiation enabled the PDT, while NLG919 reduced the ITM by suppressing the activity of IDO-1 (Fig. 4b). The combination of LINC treatment and two-wave laser irradiation (namely, LINC^L^) led to higher ROS generation efficacy than did LINC alone. In the 4T1 xenograft tumor model, LINC^L^ significantly increased the cell surface expression of CRT, promoted HMGB1 release (Fig. 4c, d), and facilitated DC maturation in the tumor-draining lymph nodes to a greater extent than were realized in the laser-insensitive group. In addition, the ratio of intratumorally infiltrating CD8^+^ T cells was higher than that in the laser-insensitive group, thus resulting in a higher level of tumor eradication without lung metastasis (Fig. 4e, f).

### Immune checkpoint blockade (ICB)

**Fig. 5:**
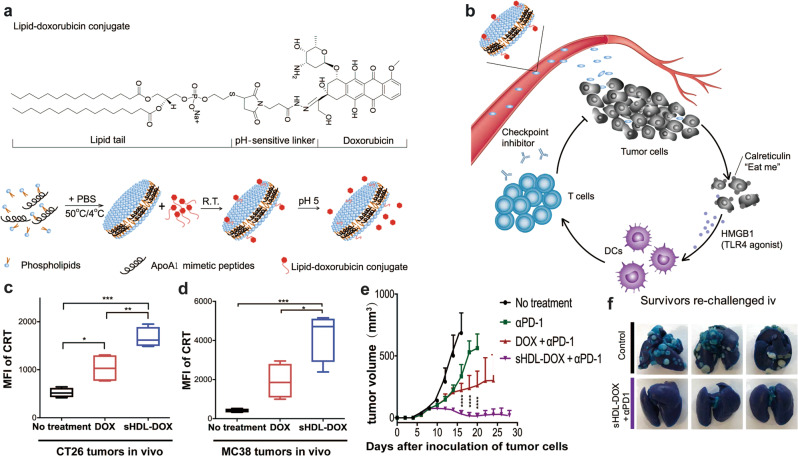
Schematic of sHDL-DOX to enhance cancer immunotherapy by combining ICD and αPD-1. **a** The chemical structure and self-assembly process of a sHDL; **b** The mechanism of sHDL-triggered ICD-mediated antitumor immunity synergized with ICB. The level of CRT cell surface expression in **c** CT26 and **d** MC38 tumor models; **e** tumor volume over time and **f** Lung metastasis in CT26 tumor-bearing mice. Adapted with permission from [91]. Copyright (2019) Science Publishing Group.

ICB therapy using either anti-programmed death 1 (αPD-1), anti-programmed death ligand 1 (αPD-L1) or anti-cytotoxic T lymphocyte-associated antigen 4 antibodies has shown potential in the clinic [89, 90]. Thus, combination ICB therapy using nanomedicine strategies is a promising approach. To promote immunogenicity and combat ITM simultaneously, high-density lipoprotein nanodiscs (sHDLs) composed of DOX, phospholipids and the apolipoprotein A1 mimetic peptide were designed (Fig. 5a, b) [91]. These sHDLs displayed prolonged blood circulation and tumor-specific accumulation of DOX that enhanced the release of HMGB1 and promoted cell surface expression of CRT in both CT26 and MC38 tumor models (Fig. 5c, d). Antitumor studies in vivo demonstrated that the combination therapy of sHDL and αPD-1 enhanced the frequency of intratumoral infiltrating CTLs compared with that shown in either αPD-1 monotherapy or combination therapy with DOX and αPD-1. sHDLs eliminated most of the established tumors and prevented lung metastasis (Fig. 5e, f).

In addition to the small molecule inhibitors and monoclonal antibodies for combating ITM, peptide-based immune checkpoint inhibitors have also been exploited for enhancing ICD by ICB. For example, a biomimetic nanoparticle has been designed to integrate PPa, a ROS-activated PTX prodrug, and αPD-L1 peptide (dPPA) into a single nanoplatform [92]. Combination immunotherapy with dPPA simultaneously enhanced tumor immunogenicity by inducing ICD and attenuated the ITM by blocking PD-L1, resulting in significant tumor regression.

CD47 is another crucial immune checkpoint protein that is abundantly expressed on the surface of various tumor cells. To prevent phagocytosis of tumor cells by APCs, CD47 acts as a “don’t eat me” signal by binding with signal regulatory protein-α (SIRP-α) on the surface of macrophages or DCs [93]. Therefore, CD47 suppresses the ICD effect by inhibiting antigen uptake by APCs. To address this challenge, a nanocage based on engineered human ferritin was designed to codeliver an SIRP-α variant and DOX (termed HFSIRP-α-DOX) [94]. The combination of ICD induction and CD47 blockade by HFSIRP-α-DOX provoked a long-term antitumor immune response. The in vivo antitumor studies showed that HFSIRP-α-DOX significantly increased the intratumoral infiltration of CD8^+^ T cells compared with that of the control group. HFSIRP-α-DOX efficiently suppressed tumor growth by activating the adaptive immune response and combating the ITM.

To further demonstrate the potential of ICD induction and CD47 blockade for combination immunotherapy, a tumor microenvironment-activatable nanoparticle was designed to destroy tumor cells and induce ICD. The nanoparticles were constructed from an MMP-2-activatable vesicle (MPV) and 2,3-dimethylmaleic anhydride-modified hexadecyl-oxaliplatindiethylene amine (HOAD), which was termed MPV-HOAD [95]. The MPV-HOAD vesicles specifically accumulated in tumors and were activated by tumor acidity and MMP-2/9. The combination of MPV-HOAD with NIR laser irradiation induced the immunogenic death of tumor cells in the CT26 tumor xenograft model, while the addition of an intratumoral injection of αCD47 was most effective at suppressing tumor growth.

## Conclusion and future perspectives

In summary, several emerging therapeutic methods, including chemotherapy, phototherapy, and radiotherapy, have been shown to be effective for eliciting ICD. However, ICD-activated immune responses only moderately suppressed tumor recurrence and metastasis due to insufficient tumor distribution of these therapies. To improve the therapeutic performance of ICD-based cancer immunotherapy, nanoparticle-based drug delivery systems enable codelivery and multiple irradiation regimens to enhance the performance of ICD-based therapies. The combination of nanotechnology and ICD-based cancer immunotherapy shows potential for improved cancer immunotherapy by simultaneously eliciting antitumor immunity and suppressing the ITM.

Despite great success using these nanoparticle-based ICD therapies in preclinical studies, their clinical application is still hindered by several challenges. First, the nanoparticle-based ICD strategy is in its infancy, with many novel tactics remaining to be explored. Tumor-specific drug distribution and activation are crucial for eliciting antitumor immunity. Second, simple nanoparticle designs and quality control mechanisms are essential for the clinical translation of nanomedicine-based cancer immunotherapy. Third, further efforts to elucidate the mechanisms underlying ICD-based cancer immunotherapy are required to facilitate its clinical translation.
